# Mutation survey of the optic atrophy 1 gene in 193 Chinese families with suspected hereditary optic neuropathy

**Published:** 2013-02-06

**Authors:** Yabin Chen, Xiaoyun Jia, Panfeng Wang, Xueshan Xiao, Shiqiang Li, Xiangming Guo, Qingjiong Zhang

**Affiliations:** State Key Laboratory of Ophthalmology, Zhongshan Ophthalmic Center, Sun Yat-sen University, Guangzhou 510060, China

## Abstract

**Purpose:**

Dominant optic atrophy (DOA) is the most common form of autosomal inherited optic neuropathy, mainly caused by mutations in the optic atrophy 1 (*OPA1*) gene. The purpose of this study was to detect *OPA1* gene mutations and associated phenotypes in Chinese patients with suspected hereditary optic neuropathy.

**Methods:**

A cohort of 193 Chinese families with suspected hereditary optic neuropathy was collected, which had been excluded from the three common primary mitochondrial DNA mutations associated with Leber hereditary optic neuropathy in our prior screening. Sanger sequencing was used to analyze variants in the coding and adjacent regions of *OPA1*.

**Results:**

In this study, 11 heterozygous *OPA1* mutations, among which eight were novel and three were known, were identified in 12 of the 193 families (6.2%) but in none of the 192 control individuals. These novel mutations consisted of two nonsense mutations (p.E707* and p.K797*), two missense mutations (p.T330S and p.V377I), two deletions (p.S64fs and p.L331fs), one small insertion (p.L17fs), and one splice site mutation (c.2614–2A>G). Of the 12 families, three had a family history of optic neuropathy while nine were sporadic cases. Analysis of the family members in the two sporadic cases demonstrated that one parent in each of the two families had the *OPA1* mutation and mild phenotype of optic atrophy. A 4-year-old boy with severe ocular phenotype was found to be compound heterozygous for two *OPA1* mutations, a p.S64fs frameshift deletion and a p.V377I missense mutation, possibly implying an additive effect.

**Conclusions:**

This study implies that the frequency of DOA is much lower than that of Leber hereditary optic neuropathy in Chinese compared with other ethnic groups. Lack of awareness of the mild phenotype of DOA may contribute to the low frequency of *OPA1*-related DOA in Chinese. The phenotype associated with compound heterozygous *OPA1* mutations may suggest a possible addictive effect.

## Introduction

Optic neuropathy, a common visual impairment, can be caused by genetic defects, toxic factors, and ocular or brain diseases [1-3]. Genetic defects may occur in nuclear genome or mitochondrial DNA (mtDNA) [4-6]. Mutations in mtDNA are known to cause Leber hereditary optic neuropathy (LHON, MIM 535000) [7]. At least eight loci (OPA1 to OPA8) have been mapped to the nuclear genome in association with optic atrophy, in which three causative genes have been identified, including the optic atrophy 1 (*OPA1*, OMIM 605290) gene, the optic atrophy 3 (*OPA3*, OMIM 606580) gene, and the transmembrane protein 126A (*TMEM126A*, OMIM 612988) gene [1].

Dominant optic atrophy (DOA, MIM 165500) is the most common form of hereditary optic neuropathy resulting from mutations in nuclear genome, with a prevalence of 1 in 50,000 overall [8] and as high as 1 in 35,000 in the north of England [9], similar to the frequency of LHON (1 in 25,000 to 1 in 50,000) as reported in Caucasians [1,10]. DOA typically presents as painless and slowly progressive visual loss, occurring insidiously with a mean age of onset at 6–10 years of age [1,11]. DOA exhibits marked inter- and intrafamilial variable phenotypes [9,12,13]; for instance, about 25% of patients were found to have optic atrophy with fundus examination but without a complaint of visual deterioration [11,14].

Of the three nuclear genes known to cause optic neuropathy when mutated, mutations in *OPA3* and *TMEM126A* are extremely rare. *OPA3* mutations result in optic atrophy associated with cataract [15,16], while *TMEM126A* mutations are responsible for an autosomal recessive form of optic atrophy [17,18]. Mutations in *OPA1* account for the majority (60%–70%) of patients with DOA [19]. Thus far, at least 230 pathogenic mutations have been identified in *OPA1* [20]. However, extremely variable phenotypes of DOA hindered the diagnosis and, therefore, the clinical test of *OPA1* mutational screening. Until now, the genotype-phenotype correlations of *OPA1* mutations with DOA were elusive [21,22]. Recently, analysis of *OPA1* in some large case series of hereditary optic neuropathy in Caucasians suggested that scanning the most frequently mutated exons might be a good strategy for identifying *OPA1* mutations [21,23]. Although exon 27, exon 8, and exon 15 may be mutational hot spots in Caucasians [21], the frequency of *OPA1* mutation in Chinese patients has not been evaluated. Only a few Chinese families with DOA have been reported to be associated with *OPA1* mutations [24-27], not to mention the mutational hot spots. In this study, Sanger sequencing was used to detect the mutation of *OPA1* in 193 Chinese families with suspected hereditary optic neuropathy, in which the three common LHON-associated primary mutations of mtDNA had been excluded with prior screening.

## Methods

### Patients

Probands with suspected hereditary optic neuropathy from 193 unrelated families were examined at the Pediatric and Genetic Clinic in Zhongshan Ophthalmic Center. The criteria for participating in this study were as follows: (1) reduced visual acuity not related to refractive error or ocular media; (2) optic atrophy revealed with fundus examination; (3) exclusion of optic atrophy with known causes; and (4) absence of the three common primary LHON-associated mutations in mtDNA, which was confirmed based on methods described in our previous study [28]. All probands were assessed by experienced ophthalmologists or neuro-ophthalmologists. Informed consent conforming to the tenets of the Declaration of Helsinki and the Guidance of Sample Collection of Human Genetic Diseases (863-Plan) by the Ministry of Public Health of China was obtained from the participants or their guardians before the study. For the probands from the 193 families, 155 were sporadic cases, and 38 had a family history of hereditary optic neuropathy; 132 were male, and 61 were female.

### Mutation screening

Genomic DNA from the proband of each family was prepared from peripheral blood leukocytes as previously described [23]. All the coding exons and exon-intron junctions of *OPA1* (references from NCBI: NC_000003.11 for gDNA, NM_015560.1 for mRNA, and NP_056375.2 for protein) were amplified with PCR using 30 primer sets (Table 1). The primer pairs were designed to cover at least 80 bp of each exon-intron junction, aiming to detect most of the known intronic mutations. PCR reaction was performed in 20 μl volumes containing 80 ng genomic DNA. Touchdown PCR was performed, which consisted of a denaturizing step at 95 °C for 5 min and annealing temperature decreased by 2 °C after the first 5 cycles and the second 5 cycles, and then down to the optimal annealing temperature (listed in Table 1), and a final extension at 72 °C for 5 min. Sequences of the amplicons were determined with BigDye Terminator cycle sequencing kit v3.1 and an ABI3130 Genetic Analyzer (Applied Biosystems, Foster City, CA). Sequences from patients and the *OPA1* reference sequence (NM_015560.1) were compared using SeqMan II software (Lasergene 8.0, DNASTAR, Madison, WI). All variations were confirmed with bidirectional sequencing, and novel mutations were then evaluated in 384 chromosomes of 192 normal individuals. The effect of a novel missense mutation on the encoded protein was predicted with the Blosum62 Table [29] as well as PolyPhen-2 [30] and the SIFT online tool [31]. Splice site mutations were predicted with the Splice Site Prediction by Neural Network [32]. In addition, the degree of evolutionary conservation at amino acid positions altered by novel *OPA1* mutations was analyzed using the MegAlign program of the Lasergene package. Segregation analyses of mutations were performed on patients with available family members.

**Table 1:** Primers used to amplify the genomic fragments of *OPA1*.

Fragment	Forward primer (5′-3′)	Reverse primer (5′-3′)	Product length (bp)	Annealing temperature
Exon 1	CCTCGGCCGCGGCTCTGTGC	GGGCTCCTGTCATTCTGGGTCCTCAAG	327	65 °C
Exon 2	TTTGCACCACATTTTCCTCATCT	GGCATCTTCCTATTAGCATCATTA	743	60 °C
Exon 3	GGGCAAAATTATGAAACCT	TAAAATTATGGGCTACTGG	523	57 °C
Exon 4	ACTGCCTGGGCTGGAAC	GGAACTGTCATTTTACTGGGC	507	65 °C
Exon 4b	GCCCTATCGTAATATGAAATCTGAG	GCATAAGCAGCATTATAAATTTGG	257	60 °C
Exon 5	AGGCGATTTGATTCTTTGA	ACTTGGATGTTTTTGTATTTG	320	55 °C
Exon 5b	AACCATCCCTCCCTAGCTTACATCT	GGCTTTACCTATACTACCCACTCCAG	396	65 °C
Exon 6	CTTTCATAAGAAATGACTAGAATAGCAACA	TGGGCATAAGATTCACTCAAAATAGG	560	57 °C
Exon 7	ATGTGAGTAGCAAGGAATTTTCCAAGTG	CCTCCAAGCACATTAGGTTAGAAAGAAA	484	65 °C
Exon 8	CTAAATAAACTGAATGAGAAATGGAC	ACATTACTTGGAACATGTAAGATTAC	446	60 °C
Exon 9	GTTTTGCTGTTCCTATTTTCAATGTGCA	GCCTCCTGGCTGTGCCTTCTACTGATTT	464	65 °C
Exon 10–11	CCCAGCAGTAGTGTGAAGGG	AAAACAATGCTAAAGTTTGGGG	716	60 °C
Exon 12–13	TGTGAGCGTCTTATCTGAATGGA	AATGAATACGAAGAGAAGGCAAAAA	506	60 °C
Exon 14	TTGCTATAATGTAGACACAGGGG	CCATGTACCATTTCCTTTTGTG	395	60 °C
Exon 15–16	TCATTCCGGGTTTTCGATAC	AAACTGCTCTCAATTCTGCC	662	65 °C
Exon 17	GCTACCGTATTGGAATGTTTTCCTCCTC	AAATGTTCTCATCTGTTTGAACTCTGCA	525	65 °C
Exon 18	GGGTCATAGGCGCACTCTC	TCTCAGAAAACACTTTCAACACTTG	425	57 °C
Exon 19	CCCAAATTCAGCCTAGTCAAAAA	GAGCCAAGGCAACAATAAATCAC	293	65 °C
Exon 20	GCTGGAGTGGAAGAACAAAGACAAA	CCCAAAACAGAGATGAGGAATAAAGAA	616	65 °C
Exon 21	CCATATCTGTCCCCAGCAAC	GCAACAGGTGATTTTAGAAGGG	541	65 °C
Exon 22	TAACAAATAAGCAGGCAAGTAAAAGAAG	CATTGGTTTTAGTAGTTACAAAGCAGTT	464	60 °C
Exon 23	ATGTGGGTTTTTTCCTTTA	GCATGTTTCATCTCTTGTC	448	57 °C
Exon 24	TGATTAAGCTTGTGTTATCTTTTATGC	CGTGACAAAAGTCAAATTAAGCAC	372	60 °C
Exon 25	CCTACCCTGTCTACTCCACAAG	TTTCCCCAGATGATCAAAGG	523	60 °C
Exon 26	TTAAGCTTAGGACATATCTACTGGTTC	TGGGAAGTATTTTGGCATCC	291	60 °C
Exon 27	TTTTGGGAAATCTGCACTTC	TCTGACCTTGTTTTCCACCC	533	60 °C
Exon 28	TTGGGTAAAAGGTGGTATGGTGAG	CAAGCAGGATGTAAATGAAGCAGAA	309	65 °C

## Results

### Optic atrophy 1 mutations

**Figure 1:**
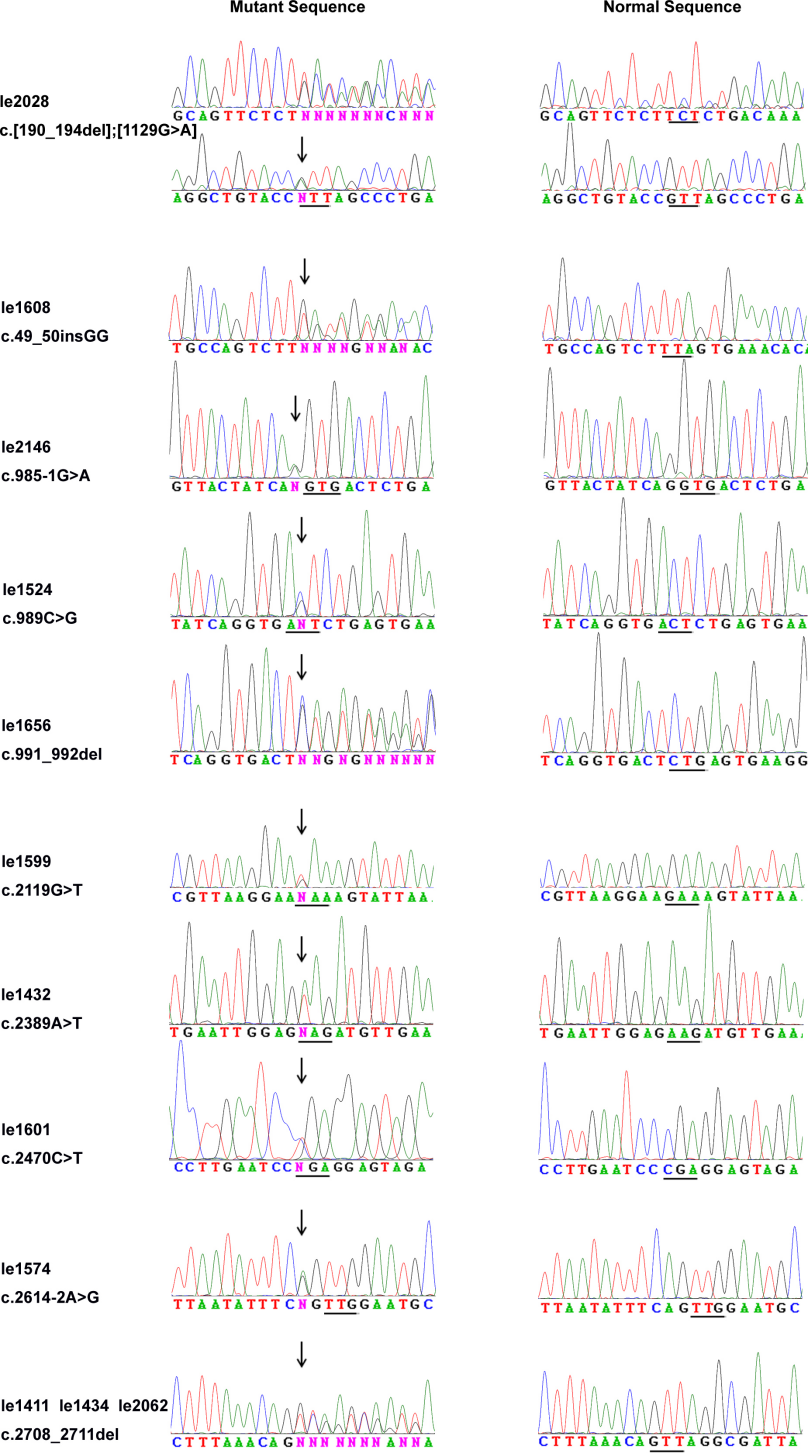
Sequence chromatograms. The 11 sequence changes detected in the probands with dominant optic atrophy are shown (left column) compared with corresponding normal sequences (right column). The mutational sites are indicated with an arrow, and the amino acid codes are depicted with a line.

Upon complete sequencing analysis of *OPA1* coding exons and adjacent intronic regions, 11 heterozygous mutations in *OPA1* (Table 2, Figure 1) were detected in 12 of 193 (6.2%) families, among which eight were novel and three were known. These mutations consisted of three nonsense mutations, two splice site mutations, three deletions, two missense mutations, and one small insertion. The c.2708_2711del mutation in exon 27 was present in three of the 12 probands (25%). In addition, three mutations were found in a fragment less than 10 bp around the 5′ end of exon 10. Of the 12 cases, nine were sporadic cases (9/155, 5.8%), and three had a family history (3/38, 8.0%). The eight novel mutations were predicted to be pathogenic and not detected in 384 control chromosomes. Polymorphisms detected in *OPA1* are listed in Table 3.

**Table 2:** *OPA1* mutations identified in the 12 Chinese families with optic atrophy.

OPA1	Patient ID	Nucleotide change	Amino acid change	Status	Conser vation	Computational prediction			Allele frequency in		Reported
						Blosum62	PolyPhen or Splice site	SIFT	cases	controls	
exon 2	le1608	c.49_50insGG	p.L17fs	Hetero	-	-	-	-	1/386	0/384	This study
exon 2	le2028	c.190_194del	p.S64fs	Hetero	-	-	-	-	1/386	0/384	This study
intron 9	le2146	c.985–1G>A	Splicing defect	Hetero	-	-	-	-	1/386	NA	Delettre et al. [40]
exon 10	le1524	c.989C>G	p.T330S	Hetero	Yes	5>1	PrD	D	1/386	0/384	This study
exon 10	le1656	c.991_992del	p.L331fs	Hetero	-	-	-	-	1/386	0/384	This study
exon 11	le2028	c.1129G>A	p.V377I	Hetero	Yes	4>3	PrD	T	1/386	0/384	This study
exon 21	le1599	c.2119G>T	p.E707*	Hetero	-	-	-	-	1/386	0/384	This study
exon 24	le1432	c.2389A>T	p.K797*	Hetero	-	-	-	-	1/386	0/384	This study
exon 24	le1601	c.2470C>T	p.R824*	Hetero	-	-	-	-	1/386	NA	Ferre et al. [21]
intron 25	le1574	c.2614–2A>G	Splicing defect	Hetero	-	-	SSA	-	1/386	0/384	This study
exon 27	le1411, le1434, le2062	c.2708_2711del	p.V903fs	Hetero	-	-	-	-	3/386	NA	Ferre et al. [21]

**Figure 2:**
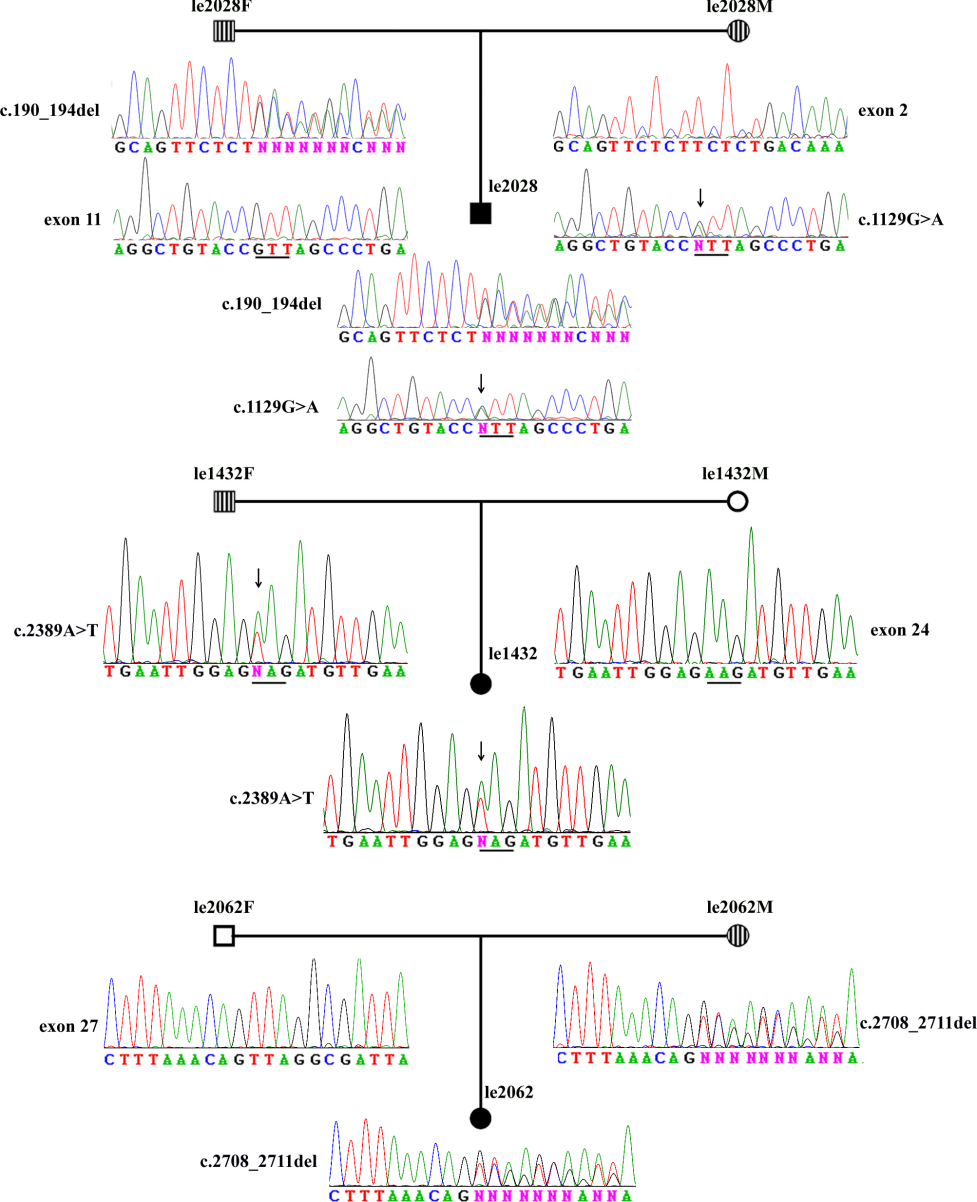
Segregation analysis of Optic Atrophy 1 mutations in three families with dominant optic atrophy. Circles represent women, and squares represent men. Two circles and a square filled in black indicate probands with suspected hereditary optic atrophy. Circles and squares filled with lines show carriers with Optic Atrophy 1 (*OPA1*) variants. Proband le2028 is compound heterozygous for *OPA1* mutations. The father has a c.190_194del mutation in exon 2, and the mother has a c.1129G>A in exon 11. Both mutations transmitted to their son. Proband le1432 inherited a c.2389A>G mutation from her father. Proband le2062 inherited a c.2708_2711del mutation from her mother. The mutational sites are indicated with an asterisk, and amino acid codes are depicted with a line.

The proband le2028 is compound heterozygous for two *OPA1* mutations, a frameshift deletion and a missense mutation (Figure 2). Abbreviations: Hetero, Heterozygous; PrD, probably damaging; SSA, splicing site abolished; D, damaging; T, tolerated; NA, not analyzed.

**Table 3:** Polymorphisms detected in the study.

Nucleotide change	Amino acid change	Status	Conser- vation	Computational prediction			Allele frequency in cases	Reported or SNP ID
				Blosum62	PolyPhen or Splice site	SIFT		
c.43C>A	p.Q15K	Hetero	No	5>1	B	T	1/386	rs75414918
c.321G>A	p.(S107=)	Hetero	NA	-	NC	-	1/386	rs117888848
c.473G>A	p.S158N	Hetero/homo	No	4>1	B	T	125/386	rs7624750
c.557–19T>C	-	Hetero/homo	NA	-	NC	-	109/386	rs3772393
c.575C>T	p.A192V	Hetero	Yes	4>0	B	T	9/386	rs34307082
c.624+13G>C	-	Hetero	NA	-	NC	-	3/386	this study
c.625–4T>A	-	Hetero	NA	-	NC	-	1/386	this study
c.870+4T>C	-	Hetero/homo	NA	-	NC	-	12/386	Liu Y et al. [41,42]
c.870+32T>C	-	Hetero/homo	NA	-	NC	-	105/386	Liu Y et al. [41,42]
c.871–9T>A	-	Hetero	NA	-	NC	-	1/386	this study
c.1177A>C	p.(R393=)	Hetero	NA	-	NC	-	1/386	rs149752576
c.1608A>C	p.(A536=)	Hetero	NA	-	NC	-	11/386	rs78767626
c.1770+16T>G	-	Hetero/homo	NA	-	NC	-	27/386	rs9831772
c.1884A>G	p.(V628=)	Hetero	NA	-	NC	-	9/386	rs73069703
c.1923G>A	p.(A641=)	Hetero	NA	-	NC	-	3/386	rs138114609
c.2109T>C	p.(A703=)	Hetero/homo	NA	-	NC	-	121/386	rs9851685
c.2276–128_2276–127insT	-	Hetero	NA	-	NC	-	1/386	this study
c.2808G>A	p.(A936=)	Hetero/homo	NA	-	NC	-	35/386	rs117475774
c.2796C>T	p.(R932=)	Hetero	NA	-	NC	-	8/386	rs35540805
c.2819–4A>G	-	Hetero	NA	-	NC	-	2/386	rs184273607
c.2883+2T>C	-	Hetero	NA	-	NC	-	1/386	this study

Abbreviations: Hetero, Heterozygous; Homo, Homozygous; B, benign; NC, not changed; T, tolerated; NA, not analyzed.

### Optic atrophy 1 compound heterozygous mutations

A 4-year-old boy harbored two *OPA1* mutations, c.190_194del (p.S64fs) and c.1129G>A (p.V377I). Segregation analysis demonstrated that the deletion was inherited from his father and the missense mutation from his mother (Figure 2), indicating compound heterozygosity. The boy was observed suffering from poor vision as early as 2 years of age. Fundus examination revealed temporal disc pallor in both eyes when he was 4 years old. Retinal nerve fiber layer (RNFL) thinning was confirmed with optical coherence tomography (OCT). Pattern visual-evoked potential (PVEP) showed prolonged latencies and diminished amplitudes in both eyes. In contrast, his father presented a much milder phenotype: His best-corrected visual acuity was 20/30 (Snellen) for both eyes, and his refraction was −1.50 sph for the right eye and −3.50 sph for the left eye. Fundus examination of the father showed bilateral disc pallor similar to his son while OCT and PVEP tests revealed milder abnormalities compared with the son. The mother had normal visual acuity of 20/20 (Snellen) for both eyes without any detectable abnormalities on fundus observation, OCT scan, and PVEP recordings. No extraocular neurologic sign presented in the proband and his parents (Table 4).

**Table 4:** Clinical characteristics of the patients with *OPA1* mutations.

Patient ID	OPA1 mutations	Sex	Age (year) at		Inheri- tance	BCVA		Optic	VEP		RNFL thinning		Scotoma	
			exam	onset		OD	OS	atrophy	prolonged latencies	diminished amplitudes	OD	OS	OD	OS
le1608	c.49_50insGG	M	12	9	Spo	0.3	0.2	Yes	Yes	No	NA	NA	NA	NA
le2028	c.[190_194del]; [1129G>A]	M	4	2	Fam	0.05	0.05	Yes	Yes	Yes	Yes	Yes	NA	NA
le2028F	c.190_194del	M	39	NA	Fam	0.6	0.7	Yes	Yes	No	Yes	Yes	No	No
le2028M	c.1129G>A	F	33	NA	Fam	1.5	1.5	No	No	No	No	No	No	No
le2146	c.985–1G>A	M	16	6	Fam	0.2	0.4	Yes	Yes	Yes	NA	NA	Yes	Yes
le1524	c.989C>G	M	17	17	Fam	0.3	0.15	Yes	NA	NA	NA	NA	Yes	Yes
le1656	c.991_992del	M	8	7	Spo	0.1	0.1	Yes	Yes	Yes	NA	NA	Yes	Yes
le1599	c.2119G>T	F	24	14	Spo	0.3	0.3	Yes	Yes	Yes	NA	NA	Yes	Yes
le1432	c.2389A>T	F	13	10	Spo	0.4	0.2	Yes	NA	NA	Yes	Yes	Yes	Yes
le1432F	c.2389A>T	M	41	NA	Spo	0.7	0.7	Yes	NA	NA	Yes	Yes	NA	NA
le1601	c.2470C>T	F	35	35*	Spo	0.3	0.2	Yes	NA	NA	NA	NA	NA	NA
le1574	c.2614–2A>G	M	24	24*	Spo	0.2	0.3	Yes	NA	NA	NA	NA	NA	NA
le1434	c.2708_2711del	M	6	3	Spo	0.2	0.4	Yes	Yes	Yes	Yes	Yes	NA	NA
le1411	c.2708_2711del	M	9	6	Spo	0.2	0.2	Yes	NA	NA	NA	NA	NA	NA
le2062	c.2708_2711del	F	17	12	Spo	0.4	0.4	Yes	Yes	Yes	NA	NA	No	No
le2062M	c.2708_2711del	F	49	NA	Spo	0.7	0.7	Yes	NA	NA	Yes	Yes	NA	NA

Analysis of the family members in two sporadic cases (le1432 and le2062) demonstrated that one parent in each of the two families had the *OPA1* mutation and mild phenotype of optic atrophy. The asterisk indicates the time when obvious visual deterioration occurred after taking antituberculosis medicine. Abbreviations: Fam, family; Spo, sporadic; BCVA, best-corrected visual acuity; RNFL, retinal nerve fiber layer; M, male; F, female; OD, right eye; OS, left eye; NA, not available.

### Clinical characteristics of optic atrophy 1-positive patients

All probands with *OPA1* mutations had clinical symptoms and signs of DOA (Table 4). The disease occurred insidiously in most probands, with reduced visual acuity noticed at about 12 years old (12±9.5 years; ranging between 2 and 35 years). The mean age for the probands’ first visit to the eye clinic was about 15 years (15±8.8 years; ranging from 4 to 35 years). Interestingly, two probands (le1574 and le1601) experienced significant visual deterioration after taking antituberculosis medicine. Additional segregation analysis of the mutations was performed in two sporadic cases, i.e., le1432 and le2062, through contact tracing of the *OPA1*-positive probands (Figure 2). The two patients had no siblings, but their parents were available for further analysis. All four parents were farmers and were visually asymptomatic by self-report. However, the father of le1432 and the mother of le2062 harbored an *OPA1* mutation. Ophthalmic examination of the two “healthy carriers” demonstrated mild phenotype of optic atrophy: mild reduced visual acuity, attenuated retinal vessels, temporal disc pallor, and thinning RNFL on the OCT scan (Table 4).

## Discussion

DOA and LHON are the most common hereditary optic neuropathies in the general population [4,33,34], with similar prevalence in Caucasians [1,9]. Ferre et al. reported identification of genetic defects in 440 of 980 cases (45%) with suspected hereditary optic neuropathy, including *OPA1* mutations in 295 (30%) patients, mtDNA mutations in 131 (13%) patients, and *OPA3* mutations in 14 (1.4%) patients [21]. These results suggest that *OPA1* mutations are the most common cause for patients with suspected hereditary optic atrophy. However, there is no molecular epidemiological study for DOA in Chinese populations even though there are large case series of such studies for Chinese patients with LHON [28]. It is unclear whether DOA is rare in Chinese or may not be easily recognized in clinics compared to LHON. Clinically, it may be difficult to distinguish DOA from LHON in many cases, especially in the atrophic phase [35,36]. In our previous report of a molecular epidemiological analysis of 903 families with optic neuropathy, the three primary mutations (G11778A, T14484C, and G3460A) in mtDNA were identified in 346 families (38.3%) [28]. In this study, mutations in *OPA1* were detected in only 12 of the 193 families (6.2%). Since patients with one of the three primary mtDNA mutations (G11778A, T14484C, and G3460A) were not included in the cohort, the actual detection rate of *OPA1* mutations in Chinese patients with hereditary optic neuropathy should be even lower, indicating a significantly lower frequency of *OPA1* mutations (<6.2%) and a comparatively higher frequency of mtDNA mutations (38%) in Chinese patients with suspected hereditary optic neuropathy compared with French Caucasians (30% for OPA1 and 13% for mtDNA) [21]. Lack of awareness of the mild phenotype of DOA may contribute to the low frequency of *OPA1*-related DOA in Chinese and relatively high frequency of *OPA1* mutations in sporadic cases, which is implied by the segregation analysis of mutations in the family of le1432 and le2062. A family history showing autosomal dominant inheritance is a key indicator leading to clinical diagnosis of DOA and genetic analysis of OPA1. For the probands from the 12 families with *OPA1* mutations identified in this study, however, most (9/12, 75%) were sporadic cases (nine out of 155 sporadic cases). Similar frequency of *OPA1* mutations in singleton cases was also mentioned in other studies [16,21,24,37]. Since individuals who harbor *OPA1* mutations may have mild phenotypes that the individuals are unaware of, it is important to keep *OPA1* in mind for patients with suspected hereditary optic neuropathy without a family history and evaluate the family members of these singleton cases carefully. Moreover, such evaluation and genetic consultation to family members may be crucial since more severe phenotypes might be induced by toxic materials, such as alcohol, smoking, and some drugs, as seen in two patients who had taken medicine for tuberculosis in this study.

Although *OPA1* mutations contribute to the most (60%–70%) cases with DOA [19], the genetic diagnosis of DOA is still a challenge. *OPA1* mutations are spread over 30 exons [21]. In addition, about a quarter of the mutations are located in the intronic regions adjacent to exon-intron boundaries, which usually affect splicing [20]. Under this circumstance, a cost-effective method for identifying mutations responsible for DOA is of great value. Screening the most frequently mutated exons of OPA1 initially would be a feasible strategy. Although exons 27, 8, and 15 were suggested to be regions of mutation hot spots in a previous study by Ferre et al. [21], we are unable to suggest ethnic-specific mutation hot spots based on limited number of mutations identified in our cohort of Chinese patients. The c.2708_2711del mutation in exon 27 was present in approximately 27% of families in previous studies. The same mutation was found in three probands in our study, which accounts for 25% among the *OPA1*-positive cases, further confirming that it is a mutational hot spot [21,24,37]. Interestingly, three novel mutations, discovered in three unrelated patients, arose from a region less than 10 bp around the 5′ end of exon 10, which may imply another mutational hot spot. Thus far, this is the largest series of patients screened for *OPA1* in an Asian population. As our results provide evidence of ethnic variations in the mutation frequency of *OPA1*, analyzing causative genes and their exons in a systematic and population-specific fashion is necessary, especially in the context of genetic counseling for different ethnic groups.

To our knowledge, the patient with confirmed compound heterozygosity of *OPA1* mutations in our study is the fourth case reported so far [38,39], which represents a rare event. All cases with compound heterozygous *OPA1* mutations reported thus far had more severe phenotypes than their parents with a single heterozygous *OPA1* mutation, which may suggest an additive effect [39]. Although the mother is a possible case of non-penetrance in optic atrophy, the c.1129G>A (p.V377I) mutation is still likely to be pathogenic. First, the mutation is rare as it is not found in 384 control chromosomes or the Single Nucleotide Polymorphism database. Second, the mutation is located in an evolutionarily conserved region and is predicted to be probably damaging by PolyPhen-2. Third, the severity of the phenotype of the son could, at least partly, reflect the addictive effect of the mutation from his mother. However, it is not the only interpretation of this compound heterozygous case. A c.190_194del (p.S64fs) mutation was identified in the father, presumably causing premature termination. However, the phenotype of the father was much milder than the other probands with frameshift deletions or nonsense mutations in our study. This may suggest the existence of modifier alleles at other loci. If the son did not inherit the beneficial modifier alleles from his father, this could also result in the severity of the son’s phenotype. The effect of modifier alleles may also explain the marked intrafamilial phenotypical heterozygosity between the probands and other mutation carriers, as shown in le1432 and le2062.

In summary, this study implies that the frequency of DOA is much lower than that of LHON in Chinese compared with other ethnic groups. Lack of awareness of the mild phenotype of DOA may contribute to the low frequency of *OPA1*-related DOA in Chinese. Further analysis of *OPA1* in individuals with mild visual impairment and temporal disc pallor may be helpful in disclosing the real frequency of DOA in Chinese. Routine clinical test of *OPA1* variations in such cases may also enhance the care of eyes through consultation and pretreatment, especially for individuals who are unaware of visual problems but harbor *OPA1* mutations.
